# Tailoring Microstructure and Mechanical Properties of Additively-Manufactured Ti6Al4V Using Post Processing

**DOI:** 10.3390/ma14030658

**Published:** 2021-01-31

**Authors:** Yaron Itay Ganor, Eitan Tiferet, Sven C. Vogel, Donald W. Brown, Michael Chonin, Asaf Pesach, Amir Hajaj, Andrey Garkun, Shmuel Samuha, Roni Z. Shneck, Ori Yeheskel

**Affiliations:** 1 Nuclear Research Center-Negev, P.O. Box 9001, Beer-Sheva 84190, Israel; tiferete@gmail.com (E.T.); asafps@yahoo.com (A.P.); amirhajaj1976@gmail.com (A.H.); samuha@post.bgu.ac.il (S.S.); 2 Rotem Industries, Additive Manufacturing Center, Rotem Industrial Park, Mishor Yamin, D.N Arava 86800, Israel; michaelc@rotemi.co.il; 3 Department of Materials Engineering, Ben Gurion University, Beer-Sheva 8455902, Israel; roni@bgu.ac.il; 4 Los Alamos National Laboratory, Materials Science & Technology, MST 8, Los Alamos, NM 87544, USA; sven@lanl.gov (S.C.V.); dbrown@lanl.gov (D.W.B.); 5 Israel Institute of Metals, Technion R&D Foundation, Technion City, Haifa 32000, Israel; agar@trdf.technion.ac.il; 6 Materials Consultant, P.O. Box 7010, Shoham 6081668, Israel

**Keywords:** electron beam melting, microstructure, mechanical properties, HIP, fatigue, neutron diffraction, Ti-6Al-4V

## Abstract

Additively-manufactured Ti-6Al-4V (Ti64) exhibits high strength but in some cases inferior elongation to those of conventionally manufactured materials. Post-processing of additively manufactured Ti64 components is investigated to modify the mechanical properties for specific applications while still utilizing the benefits of the additive manufacturing process. The mechanical properties and fatigue resistance of Ti64 samples made by electron beam melting were tested in the as-built state. Several heat treatments (up to 1000 °C) were performed to study their effect on the microstructure and mechanical properties. Phase content during heating was tested with high reliability by neutron diffraction at Los Alamos National Laboratory. Two different hot isostatic pressings (HIP) cycles were tested, one at low temperature (780 °C), the other is at the standard temperature (920 °C). The results show that lowering the HIP holding temperature retains the fine microstructure (~1% β phase) and the 0.2% proof stress of the as-built samples (1038 MPa), but gives rise to higher elongation (~14%) and better fatigue life. The material subjected to a higher HIP temperature had a coarser microstructure, more residual β phase (~2% difference), displayed slightly lower Vickers hardness (~15 HV_10N_), 0.2% proof stress (~60 MPa) and ultimate stresses (~40 MPa) than the material HIP’ed at 780 °C, but had superior elongation (~6%) and fatigue resistance. Heat treatment at 1000 °C entirely altered the microstructure (~7% β phase), yield elongation of 13.7% but decrease the 0.2% proof-stress to 927 MPa. The results of the HIP at 780 °C imply it would be beneficial to lower the standard ASTM HIP temperature for Ti6Al4V additively manufactured by electron beam melting.

## 1. Introduction

Titanium alloys, especially Ti-6Al-4V )Ti64(, are widely used in the automotive [1,2,3], aerospace and biomedical industry for their high strength to weight ratio and biocompatibility. In equilibrium, at ambient conditions, Ti64 is a dual-phase material; primary phase α-Ti (HCP) co-exists with β-Ti (BCC). Increasing utilization of additive manufacturing (AM), enables the fabrication of stronger and lighter parts with more intricate geometries. AM processes yield parts with high density, commonly higher than 99.5% of the theoretical density [4,5]. Two main AM methodologies exist, the addition of material by melting wire or sprayed powder, or melting powder laid on a base plate in a layer-by-layer fashion, known as the powder bed fusion (PBF) method. Melting of powder in PBF is done either by a focused laser beam (L-PBF) or by electron beam melting (EBM). Detailed descriptions of these methods are given elsewhere [3,5,6,7] including the layout of an EBM machine [3,5,6]. There are two main differences between L-PBF and EBM; the atmosphere during the built process and the temperature of the base plate. The atmosphere during EBM is a vacuum and in L-PBF is an inert gas, typically Argon. In L-PBF of Ti64 the bed temperature is typically below 250 °C, whereas in EBM the chamber is maintained at higher temperatures [8,9,10,11], in the range of 625–700 °C [12].

In recent years, the general practice to stress relieve parts produced by L-PBF before being put to service [5,13], whereas this step is not necessary for EBM produced parts. This was attributed to the higher bed temperature in EBM, which affected the formation of a martensitic α phase. The phase diagram of Ti64 shows two solid phases α, β and a liquid phase [3]. For high cooling rates of Ti64 the β phase has a slightly different c/a ratio and the fine acicular martensite is designated as α′ [14]. This grain morphology of α′ is found in both as-built laser printed Ti64 [14,15] and in EBM parts [12,13,16,17,18]. The transformation of the α′ morphology to α + β was explored by Galarraga et al. [13]. The transition temperature of the β phase for Ti-Al-V alloys varies with the vanadium content [19,20,21] and for 4 wt % V it is between 980 °C [19] and 995 °C [13,14].

It is well known that heat treatments alter the microstructure and affect the mechanical properties of conventionally produced Ti64 [2] components. For L-PBM that underwent series of heat treatments with a variety of cooling conditions, the main findings are that temperature is the primary parameter that affects mechanical properties. Treatments between 540 °C and 1020 °C led to a monotonic increase in elongation from 5.4 ± 2.0% to 14.0 ± 2.5%, respectively, accompanied by a monotonic decrease in strength, e.g., yield stress (0.2% proof stress) between 1118 ± 39 MPa and 760 ± 19 MPa, respectively [14]. The optimum post-treatment reported was at 850 °C for two hours, followed by furnace cooling which led to a yield stress of 955 ± 6 MPa and ductility of 12.84 ± 1.36%, as compared to 1110 ± 9 MPa and 7.28 ± 1.12% in the as-built samples, respectively. After heat treatments at 780 °C the microstructure changed from martensitic α′ in the as-built material to α + β Heat-treating at 850 °C increased the β-fraction from 13% at 780 °C to 27% at 850 °C [14]. Further post-build processing procedures were studied to improve the properties of either EBM [4,10,11,13,16] or L-PBF [22] Ti64. The main reasons for performing post-processing are reducing flaw population and stress relief with minimal coarsening of the delicate microstructure of AM parts. For both processes, it is imperative to decrease defect population both in the bulk and on the parts’ surface [4,23,24,25]. As shown by [4,16,23,24,25,26], densification improves fatigue resistance. Recently Shui, X. et al. [16] studied the effects of heat treatment and hot isostatic pressing (HIP) on the tensile strength and elongation, as well as on the fatigue of Ti64 produced by EBM. The fatigue strength of the as-built and heat-treated materials had similar behavior while the HIP samples had a much higher fatigue life. The microstructure of the as-built sample was martensitic α′ whereas the microstructure after HIP was much coarser. Similar results regarding microstructure at the as-built state and after HIP at 920 °C as well as the decrease of yield stress after HIP, were reported earlier by Al-Bermani et al. [12].

HIP may have adverse effects when applied to AM parts. During HIP, concomitant heat and pressure are applied to the material [26]. The L-PBF chambers are pumped with inert gas, which is trapped inside pores and defects and can cause thermally induced porosity (TIP) [27,28,29] that leads to a decrease of density. In EBM however, the fabrication is under low vacuum, thus HIP results in nearly complete densification, as will be discussed later. The HIP process drives densification of EBM parts, reduces surface roughness and, thus, increases fatigue resistance [12] Typical HIP pressure for metals is 100 MPa and for ceramics is 200 MPa [26,30]. Typical HIP temperature, T_HIP_, for simple metals is 0.6 T_M_ to 0.7 T_M_, where T_M_ is the melting temperature in Kelvin. The various mechanisms operating during HIP are given by [25,31]. For the final densification of AM parts, which typically have grain sizes ranging from submicrons to several microns, Coble creep (diffusion around grain boundaries) [32,33] is most effective, and the homologous HIP temperature can be lowered. For titanium and its alloys the recommended temperature according to ASTM F3001 is T_HIP_ = 920 °C [26], namely 0.61 T_M_.

The mechanical properties of dense (>99% of the theoretical density) Ti64 manufactured by both L-PBF [34] and EBM method, are mainly affected by defects [4]. Small amounts of defects like lack of fusion or porosity, drastically reduce the tensile elongation [4,34] and may cause premature failures during fatigue testing [4,16]. Although it was shown that HIP at 920 °C reduced the flaw population and increased both elongation and fatigue life, it also caused a reduction of the yield stress and the ultimate tensile stress (UTS). Since the mechanical design is based on yield stress, it is suggested to define a better heat treatment that will increase material reliability.

The objective of the current study is to propose a post-process for AM by EBM, by tailoring the microstructure, hence improve the elongation and the fatigue life with a minimal decrease in the yield strength. In order to minimize the number of fatigue experiments, only the specimens which surpassed the requirements of ASTM F2924 for tensile strength and elongation, were tested in fatigue. Standard deviations of strength, elongation and fatigue life were used as indicators for the effectiveness and uniformity of the post-treatment.

## 2. Experimental

### 2.1. Sample Preparation

A tray of 225 cylindrical specimens (15 rows by 15 columns) was printed in an Arcam A2X EBM Machine (Arcam, Mölndal, Sweden). The details of powder properties, chemical composition and printing procedure are given in [4]. Specimens with a diameter of 11 mm and length of 70 mm were milled into round tensile specimens conforming to the dimensions of the appropriate standards, tensile ASTM E8M [35] and fatigue ASTM E466/E606 [36,37]. The various specimen groups with their post-treatments are summarized in Table 1. The post treatments used here were the HIP temperature (920 °C) with a pressure of 120 MPa, 780 °C with a pressure of 120 MPa in a laboratory-sized HIP apparatus. Annealing was performed in a controlled vacuum environment (10^−3^ mbar), at 780 °C and 1000 °C. Heating and cooling rates for the HIP processes were set to 4 °C/min. The heating rate in vacuum was set to 10 °C/min and specimens were furnace cooled. Dwell time in all post-treatments was two hours. Commercial samples machined from conventionally processed Ti64 were used as a benchmark for tensile tests. This material was purchased in an annealed state, and certified for ASTM F136 by the vendor. Other possible post-processes such as HIP-1000 or HT-920 were not performed, as HIP-1000 would likely lower the yield and UTS due to grain growth, and HT-920 is below the β transus temperature.

### 2.2. Sample Characterization

All specimen densities were measured using Archimedes’ method with water [38]. The volume fraction of pores, porosity, is expressed as *p* = 1 − *ρ*/*ρ*_T_, where *ρ* is the sample density and *ρ*_T_ is the theoretical density [39]. Specimens were divided into six groups, as seen in Table 1. The chemical composition is given in [4]. A Scanning electron microscope (SEM), FEI Verios XHR 460L, Berno, Czech Republic, was used for grains structure, and energy dispersive spectroscopy (EDS) (FEI Verios XHR 460L, Berno, Czech Republic) was used for chemical compositions. Electron backscatter diffraction (EBSD) (Oxford Instruments NanoAnalysis, NordlysNano, Halifax, UK ) was used for β-Ti content estimation. Neutron diffraction was performed on the HIPPO neutron time-of-flight diffractometer at LANSCE, Los Alamos, NM, USA [40,41,42,43] on three parts of a single rod. A slice of an as-built sample was extracted from the bottom, middle and top of the 70 mm long printed rod to check the homogeneity of the microstructure, i.e., texture and amount of β phase. The samples were exposed to a 10 mm diameter incident neutron beam for three sample rotations for texture measurements with count times of 5 min per rotation. The experiments were carried out between 200 °C to 1050 °C and the detailed analyses of these high-temperature runs are reported elsewhere [41]. In the present work, only the amount of β phase prior to heating in the diffractometer was desired and therefore the neutron diffraction patterns from the 45 HIPPO panels were summed for the three sample rotations collected for the texture measurements and binned into five histograms corresponding to the five nominal Bragg diffraction angles. The data, therefore, have greatly improved statistical uncertainties while averaging out the effect of the mild to moderate observed preferred orientation [41], resulting in an improved sensitivity for the beta weight fraction measurements with the Rietveld refinement method, using the General Structure Analysis System (GSAS) [43,44]. The refinement assumed structural model consisting of coexistence of both the hexagonal α-phase, assuming *P*6_3_/*mmc* space group (No. 194) with Ti, Al and V atoms randomly occupying the ‘2*c*’ site and the cubic β-phase, assuming the *lm*3*m* space group (No. 229), with the same atoms occupying the ‘2*a*’ site.

Mechanical properties of all the samples were measured along the build direction. Samples were milled to size and had a gage surface finish N6 or better. Tensile specimens from each group were tested according to ASTM E8 to determine the Young modulus, 0.2% proof stress and elongation. As a cross-check of stress–strain curves and preliminary tests to study anelasticity using neutron diffraction, selected samples were also measured at several load levels in the SMARTS neutron time-of-flight engineering diffractometer at the LANSCE facility in Los Alamos National Laboratory using neutron diffraction. The major advantage of neutron diffraction is the ability to monitor the microstructure evolution of the bulk in-situ. Neutron diffraction methodology is described in [45,46] Fatigue specimens underwent uniaxial force controlled fatigue tests (complying with ASTM E466) with stress ratio, R = 0.1 and stress, σ = 624 MPa. The frequency was 30 Hz, and the run-out (maximum number of cycles) was set at 10^7^. R = 0.1 is a common setting used in the aerospace industry, and was selected in order to allow for comparison to literature. The frequency of 30 Hz was the maximal frequency available in the machine. The stress was set at 624 MPa was selected since it is in between brittle and ductile resistance. When testing fatigue properties, many specimens are required to undergo fatigue tests, which requires much machine time as well as specimens. Since this study attempted to study fatigue properties without the use of many specimens and extended machine testing times, this stress was selected. It is better of course to test at as many stresses as possible, with as many specimens as possible in order to build a full S-N curve. Selected specimens had coupons of unstrained material removed from the tensile specimen’s bottom shoulder. The coupons were used for scanning electron microscopy, EBSD, and hardness testing. Hardness tests were performed using Vickers tester under the load of 9.807 N, HV_10N_, with a dwell time of 15 s. The results are an average of six measurements.

## 3. Results

### 3.1. Density

The average density of 130 as-built samples is 4427.8 ± 1.4 kg m^−3^ and the density of 35 samples that underwent HIP at 920 °C is 4432.0 ± 1.7 kg m^−3^ [4]. The density of 14 samples that were HIP’ed at 780 °C is 4432.0 ± 0.8 kg m^−3^. Thus, the densities following HIP at 780 °C and at 920 °C are within uncertainty, but the standard deviation is smaller at the lower temperature. The theoretical density of the current material is 4432.8 kg m^−3^ [4,6,21,47]. The relative densities of the as built and HIP samples are, 99.89% and 99.98%, respectively, i.e., the HIP process brought the relative density to within 0.02% of the theoretical density. The density of 8 samples prior and post heat treatment at 780 °C and the 6 samples treated at 1000 °C remained unchanged before and after the heat treatment, meaning pore population remained unchanged and no thermal-induced porosity was observed.

### 3.2. Phase Evolution with Temperature

**Figure 1:**
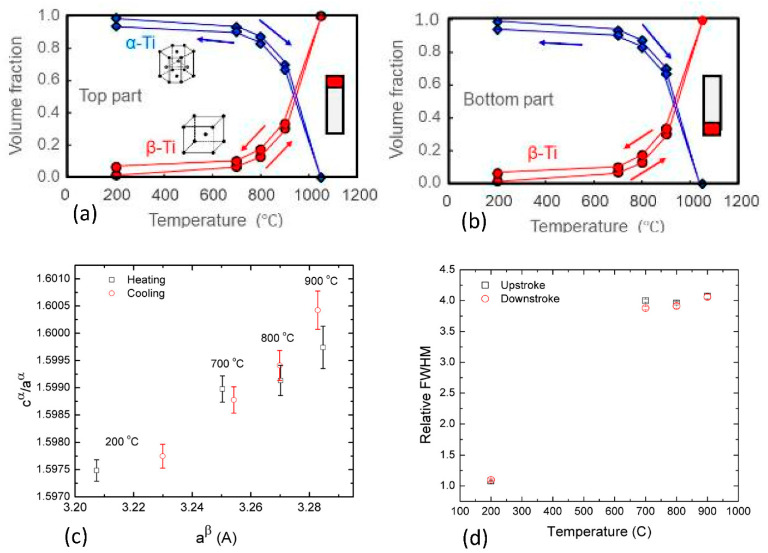
Phase change hysteresis in the top (**a**) and bottom (**b**) parts of a Ti64 rod (error bars are smaller than symbol size, measurements during heating in red and cooling in blue). The ratio between the α-phase lattice parameters c^α^/a^α^ as a function of the β-phase lattice parameter a^β^. The lattice parameters were extracted by Rietveld refinement of neutron diffraction data obtained at heating–cooling cycle of the sample (**c**). The change normalizes full width at half maximum (FWHM) of the (1011) peak of α phase as a function of temperature (**d**).

In order to study the homogeneity of the sample along its length, meaning detect if different amounts of phases evolve due to heat treatment of different parts of the printed rod, and to check if α′ exists in the as-built samples, an in-situ neutron diffraction study at LANSCE was conducted. Three slices of Ti64 taken from the bottom, middle and top of an as-built rod, were heated from room temperature (RT) to 1050 °C and cooled down to 200 °C [29,41]. The results regarding the middle part, are almost identical to those found here for the bottom and top parts [41]. Figure 1 shows the volume fraction of the phases as a function of temperature. Note that more residual β phase remains after the heat treatment. Furthermore, the transformation is not linear with temperature, as the β phase content increases rapidly with temperatures above700 °C. The differences in phase content evolution were negligible when comparing slices from the top and bottom of the same rod. From the in-situ results in Figure 1 it is possible to estimate the β volume fraction at 920 °C (in HIP-920) is about 40% and at 1000 °C (in HT-1000) it is about 70%, whereas at 780 °C (in both HIP-780 and HT-780) it is about 10%. Figure 1 also shows that upon cooling from 1050 to 200 °C some β phase retention occurs, and β content remains at ~5% after the heating and cooling cycle.

In order to reveal the effect of heat treatment on the cell parameters of the α and β phases the analysis of Xu et al. [17,18] was followed. The ratios between the refined lattice parameters of the α-phase, c^α^/a^α^, are plotted in Figure 1c as a function of the refined β-phase lattice parameter a^β^ for different temperatures. In this case, both c^α^/a^α^ and a^β^ increase with the temperature of the sample. In a recent paper c^α^/a^α^ was tested against the cell parameter of the β phase at room temperature for EBM and L-PBF Ti-6Al-4V samples [18], it is inferred that while the general trend of the EBM samples is kept for c^α^/a^α^ ratio values larger than 1.595. In contrast to the trend shown for the L-PBF samples where the decrease in c^α^/a^α^ stands for a change of the α′ phase in the as-built material to α phase after heat treatment [48]. Moreover, the relatively significant growth of a^β^ at 200 °C after the heating-cooling cycle indicates that, in addition to thermal expansion, vanadium repartitioning from the β-phase took place at high temperature and low heating rate [49].

The normalized full width at half maximum (FWHM) of the strongest diffraction peak of α phase (1011) is shown in Figure 1d as a function of temperature. Normalization was to the FWHM of this peak at room temperature. The β content at room temperature for selected samples was estimated using EBSD. The measurement procedure used has an inherent ±0.5% error, enabling a qualitative trend to be discerned. The results are detailed in Table 2, showing that as-built samples have the lowest β phase content and it increases with the post-process temperature. This indicates that while density following HIP is mildly affected by process temperature, the residual β-Ti phase concentrations increase with process temperature.

### 3.3. Microstructure

**Figure 2:**
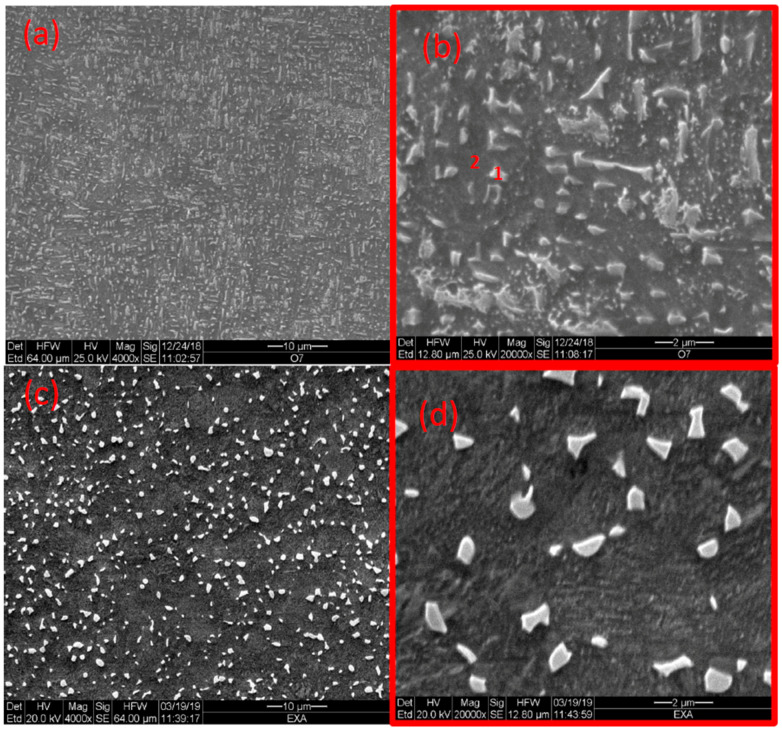
Microstructure of as-built Ti64 (**a**,**b**) and extruded commercial sample (**c**,**d**), along with EDS locations 1, 2 (1, 2 refernces the locations in the relevant table).

**Figure 3:**
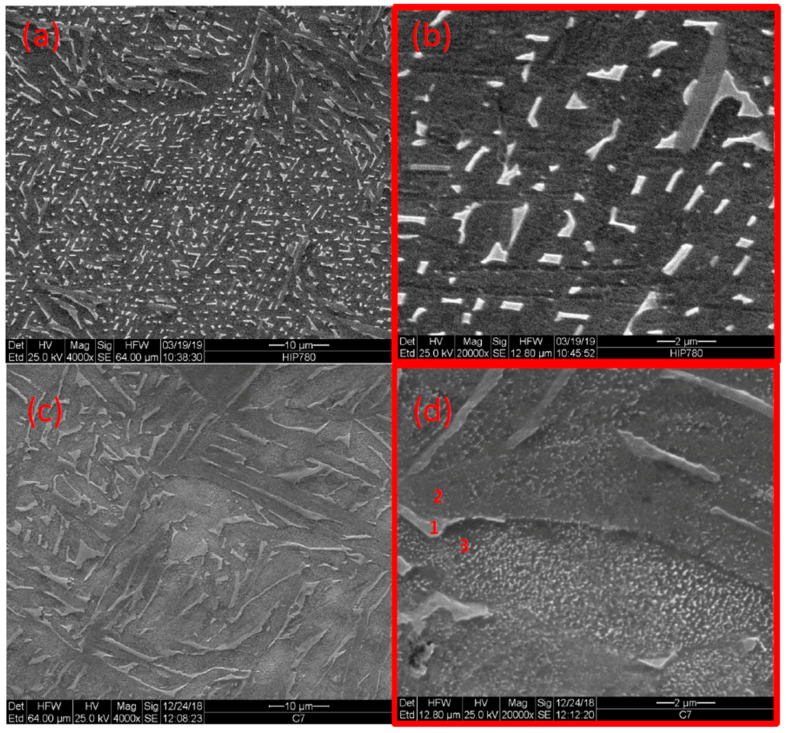
Microstructure of Ti64 after HIP-780 (**a**,**b**) and after HIP-920 (**c**,**d**) along with EDS locations 1–3 (1–3 refernces the locations in the relevant table).

**Figure 4:**
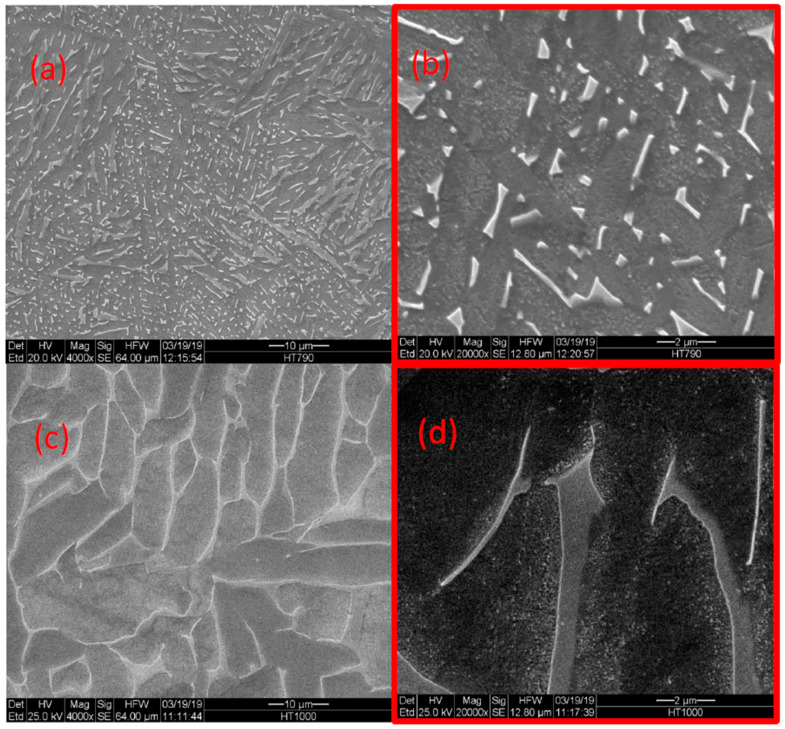
Microstructure of Ti64 after HT-780 (**a**,**b**) and after HT-1000 (**c**,**d**).

Figure 2 shows the microstructure of as-built samples as compared to extruded and annealed samples. The as-built material consists of very fine (<1 μm) acicular grains, and the latter consists of the dark phase (α) with white islands (β). Figure 3 depicts the microstructures of HIP-780 and HIP-920. Figure 4 displays the microstructure for HT-780 and HT-1000 samples. The very fine microstructure of the as-built sample was maintained in the HT-780 and HIP-780 samples, whereas the grain size in the HT-1000 and HIP-920 specimens increased. While it is difficult to measure grain size in specimens of EBM Ti64, the size and spacing of the needle-like brighter phase can be used an indication of increasing grain size [49].

An energy dispersive spectroscopy (EDS) revealed that the bright phase possesses higher vanadium content than the darker phase, typical to the β phase (Table 3). As mentioned, the β-transus temperature for Ti64 is 980 °C to 995 °C, thus the HIP-920 specimen was about 75 °C below the transus temperature, allowing more of the α phase to transform into β and coalesce into bigger grains. Indeed, the figures show that the amount of bright phase increases with the temperature of the last treatment.

### 3.4. Tensile Properties

**Figure 5:**
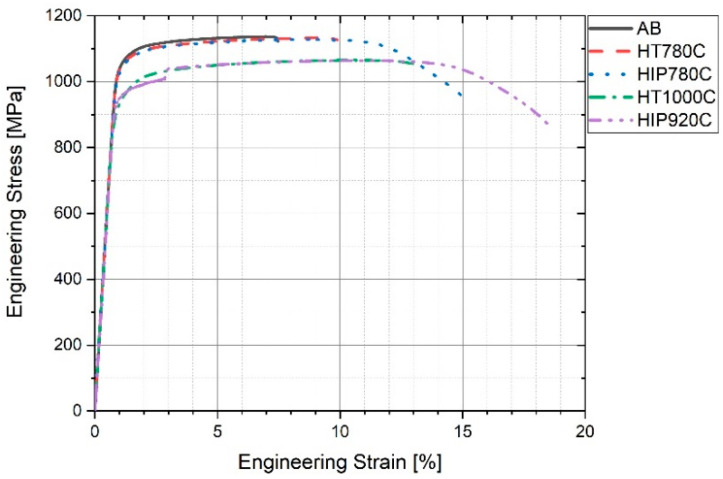
Engineering stress-strain curves of typical specimens.

**Figure 6:**
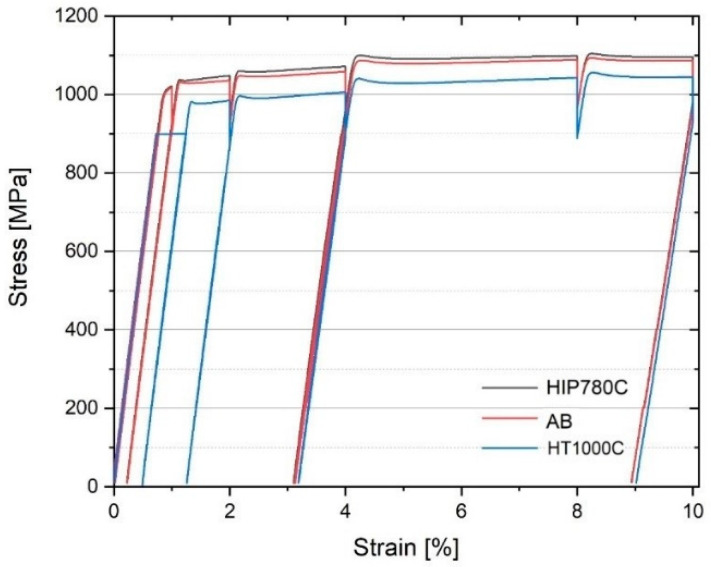
Intermittent engineering stress-strain curves of few specimens at SMARTS.

Table 4 summarizes tensile tests and hardness results. The engineering tensile stress-strain curves and the true stress-true strain curves of typical samples from the five groups are shown in Figure 5. ASTM requirements for mechanical properties of wrought and AM Ti64 are also shown. The intermittent engineering stress-strain curves of as-built, HIP-780 and HT-1000 samples are shown in Figure 6. Note that the yield stress for the as-built and HIP-780 samples are similar but the maximum stress of HIP-780 is slightly higher than that of the as-built samples. However, in Figure 6 the sample annealed at 1000 °C, crept at room temperature under a stress of 900 MPa. The tensile test results show that all the post-treatment specimens surpass ASTM mechanical properties requirements. AB refers to a group of samples from the same rows of other samples in the current study. The average yield stress values were similar to those attained from another study [4], 1036 ± 17 MPa, but the elongation here was on the lower side of the distribution of said former study, 9.8 ± 3.8%. Tiferet et al. showed the relation between specimen location and elongation [4]. As opposed to the elongation in the as-built specimens, the elongation for HIP-920 specimens increased to 20.1 ± 0.7% whereas for specimens that underwent HIP-780 the average elongation increased from 7.3 to 14.4%. The yield stress and UTS as-built and HIP-780, HT-780 are very close and higher than these properties of HIP-920 by about 60 MPa. The results show that exposure to low temperature (780 °C) does not cause any measurable changes in the strength, however, HIP treatment at the low temperature does increase the elongation of the material. Exposure to high temperature (near 1000 °C) further reduces the strength of the material and enhances the ductility.

### 3.5. Fatigue

**Figure 7:**
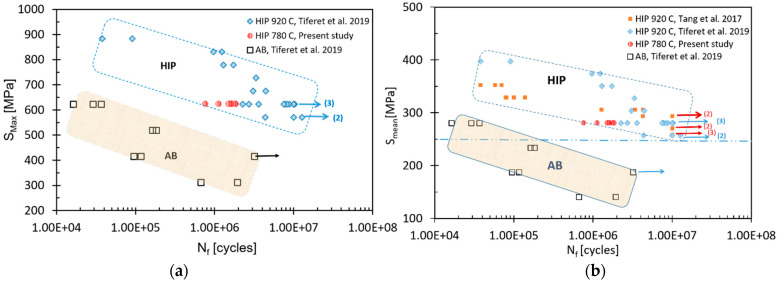
Fatigue results of the present study stress vs. the number of cycles to failure, (**a**) maximum stress (left) and (**b**) mean stress (right). For comparison, the data of Tiferet et al. [4] and of Tang et al. [50], are given. Arrows indicate runouts (1 × 10^7^ cycles) and the number of specimen runouts is summed in brackets.

**Figure 8:**
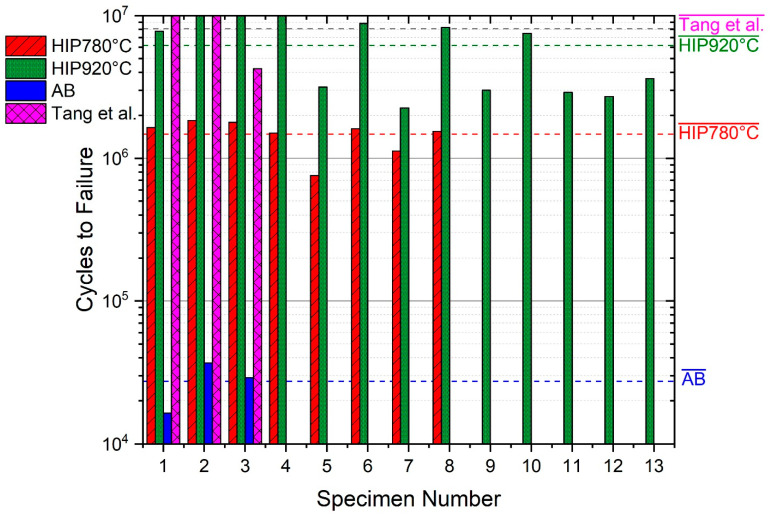
Comparative fatigue results under maximum stress of 624 MPa and R = 0.1, of AB, HIP-780, and HIP-920 specimens and of the data of Tang et al. [50] at a stress of 625 MPa; R = 0.06.

A small number of pores, lack of fusion defects, and overheating affect the tensile elongation and the fatigue life [4,34], therefore S-N curves were prepared only for the HIP specimens, since the defects in as-built or heat-treated specimens are the major locations for failure. Figure 7 shows the dramatic improvement in fatigue strength and fatigue life due to HIP in the present study as well as in other works [4,50]. Comparative fatigue test results of as-built, HIP 780 °C, and HIP 920 °C under maximum stress of ~60% yield stress, 624 ± 1 MPa and R = 0.1 are summarized in Table 5 and illustrated in Figure 8. It can be seen that performing HIP increases the number of cycles to failure by two orders of magnitude, while the HIP temperature has a lesser effect.

## 4. Discussion

Both annealing and HIP performed in this study on Ti64 produced by EBM showed increases in grain size or α phase lamellae width (Figure 3 and Figure 4). The residual β content also increases with post-treatment temperature. The high-temperature treatments led to a reduction in the hardness, yield stress and ultimate stress [18,51]. While HIP at 920 °C improves average elongation from 9.8 to 20.7%, the yield stress is reduced by about 6% (60 MPa) compared to the samples of the HIP at 780 °C. The elongation after HIP at −780 °C increased from 7.3% to 14.4%, well above the ASTM requirements. Thus, HIP at 780 °C eliminates defects (as shown by the increase in density), and improves elongation, while retaining both the finer grain structure, yield stress, and UTS. These fine grains are also important to the enhanced densification due to Coble creep. The fact that the samples’ density after HIP-780 °C is identical (within measurement error) to the density of HIP-920 °C, regarded as a standard post-treatment, proves that nearly full densification is possible using HIP at 140 °C lower temperature. The HIP-780 °C samples also exhibit smaller scatter of density, fatigue and mechanical properties as compared to the scatter of the as-built, HIP-920 °C samples (Table 4), and the scatter in data of Tang et al. [50], which may be attributed to its fine and homogeneous microstructure. It is worth noting that the mechanical properties attained after HIP at 780 °C in the current study, surpassed the mechanical properties of Ti64 fabricated recently by EBM and HIP’ed at their optimum condition, 850 °C and a pressure of 207 MPa [52].

**Figure 9:**
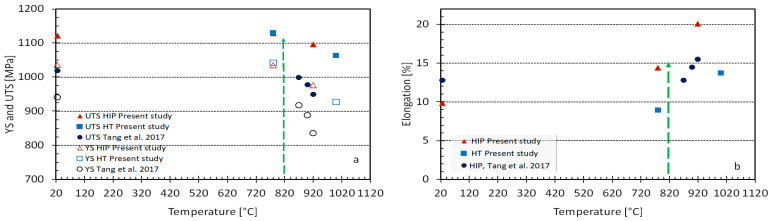
Effect of post-treatment temperature of EBM PBF additively manufactured Ti64, on the average of yield stress and ultimate tensile strength (**a**), elongation (**b**). For comparison, the data of Tang et al. [50] on HIP printed plates are also shown. Arrows indicate a better HIP process.

At the higher temperatures, the coarsening of the microstructure causes a decrease in strength, as it is well known that the yield strength of Ti64 decreases with the increase of the width of the lathes of the α phase [5,10,13,17,18,50]. Figure 9 shows the effect of post-treatment temperature for 2 h (vacuum treatment or HIP), on both the yield stress and the ultimate tensile strength. For comparison, the data of Tang et al. regarding these properties of HIP treatment of Ti64 is also plotted [47]. The increase in elongation upon heating from 780 to 1000 °C in the present study as well as for Tang data is shown in Figure 9b. From Figure 9 it is evident that although HIP at standard post-treatment, HIP at lower temperatures may result in a combination of properties that is better suited for various applications, e.g., HIP at 820 °C.

**Figure 10:**
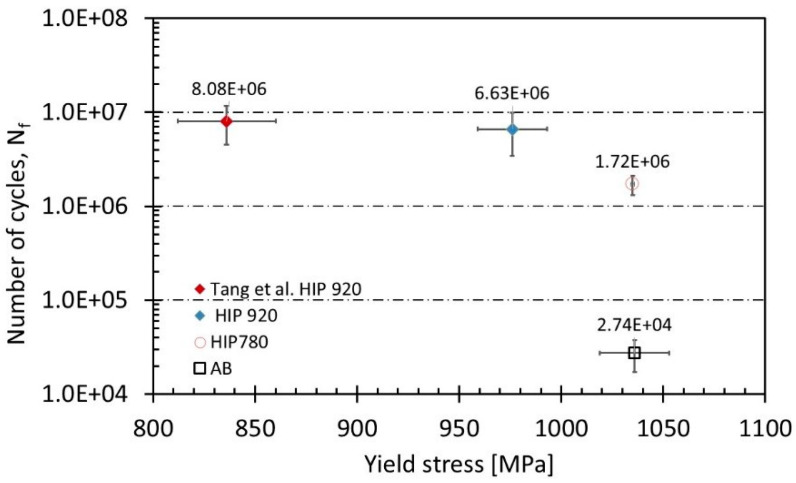
Dependence of the number of cycles to failure at constant maximum stress, 624 ± 1 MPa, on the yield stress for as-built samples and samples after various HIP treatments.

**Figure 11:**
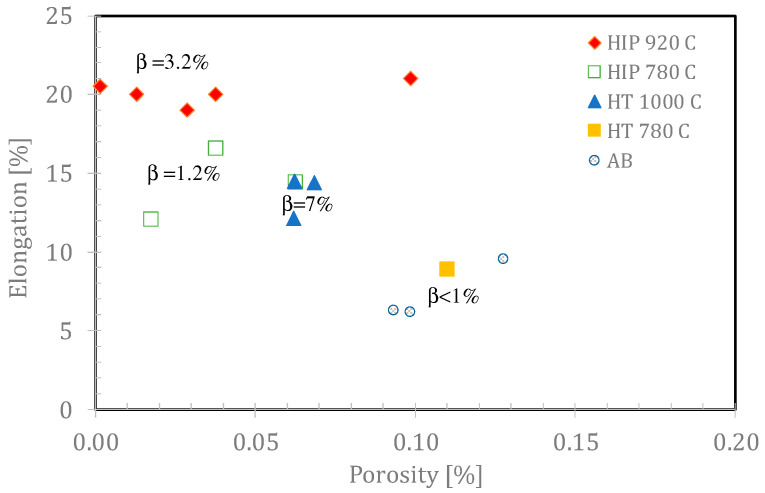
Increase in elongation with a decrease of porosity in the investigated metallurgical states. Note that the beta content also affects the elongation. The uncertainty in porosity is ±0.033%.

The fact that HIP improves the fatigue properties of Ti64 was previously reported [13,25,53,54,55]. Figure 7 shows the effect of post HIP treatments on the fatigue life under maximum stress or mean stress as compared to as-built samples. The reason for the improvement was explained mainly by the reduction of the population of lack of fusion and overheating flaws during HIP [4]. A relationship between fatigue life under constant stress, and the yield stress is shown in Figure 10. The difference in yield stress between the as-built and the HIP-780 samples is negligible but the elongation was doubled and the number of cycles to failure of HIP-780 increased by two orders of magnitudes. The fact that the difference in density between HIP-780 °C and HIP-920 °C is negligible, while both types of specimens performed much better than as-built specimens strongly supports the assumption that reduction of lack-of-fusion and overheating flaws are the root cause for the enhancement of both the elongation and the fatigue life in HIP samples. However, the presence of more β phase also might contribute to the effects. The samples HIP’ed at 920 °C exhibit higher fatigue life compared with HIP-780 °C, despite their lower yield strength (the endurance limit is usually proportional to the static strength). Finally, as to the elongation of post-treated Ti64, the increase in density (Table 2), as well as of the β phase content, led to higher elongation, as shown in Figure 11. For modifying the microstructure and properties, the density should be increased via HIP at lower temperatures and the β content can be tailored according to the continuous cooling transformation maps of Ti64 [56]. The effect of β phase content on the mechanical properties of Ti64, was shown by simulations [57,58]. It should be noted that the HT-1000 specimens also surpassed the ASTM F2924 [59] minimum requirements and thus could pose a cheaper alternative to a costly HIP process or large parts. However, as the heat treatment does not close pores or mend printing defects, it is assumed the pores and defects would greatly affect HT-1000 specimens where fatigue is concerned. However, it is suggested to perform fatigue tests for such specimens in order to determine the fatigue resistance of specimens HT1000 specimens. HT1000 seems to be a viable and cheaper replacement to HIP in certain cases, especially where large parts are concerned.

## 5. Summary and Conclusions

In this research, various post-treatments were studied in order to find a way to tailor the microstructure and the mechanical properties of EBM fabricated Ti-6Al-4V. It is demonstrated that the mechanical properties of EBM Ti64 parts change significantly as a result of post-treatments. HIP is a better post-treatment for EBM Ti64 than simple annealing under vacuum, however, the standard HIP at 920 °C recommended by ASTM, might not present the optimal treatment. HIP at 780 °C retains the fine microstructure of the as-built samples with high strength, adequate elongation, good fatigue resistance and narrow scatter of all the measured properties. Better parameters can be found by further research, e.g., based on the results in Figure 9, we recommend trying a HIP treatment at 820 °C under a pressure of 120 MPa for 2 h. The main conclusions in this study are:

1. It is possible to modify the combination of fatigue strength and other mechanical properties of AM Ti64, through changing HIP parameters.
2. HIP at 920 °C changes the fine AM microstructure due to grain growth that causes a decrease of the yield stress, YS, (976 MPa) and the UTS (1090 MPa), while HIP at 780 °C retains the fine microstructure and the high YS and UTS of the as-built samples (1038 MPa and 1135 MPa, respectively).
3. HIP improved the ductility and fatigue life due to the elimination of pores, lack of fusion and overheating flaws. HIP at 780 °C leads to nearly fully dense samples.
4. The density of HIP 780 °C vs. HIP 920 °C was identical (within measurement error), while the β phase content increased from ~1% at 780 °C to ~3% at HIP 920 °C. Thus, differences between the two HIP processes can be attributed to the changes in microstructure and β phase content and not to density.
5. HIP at lower temperature led to a small scatter of the mechanical properties due to finer microstructure. Therefore, it would be recommended, in certain applications, to use lower temperatures than the current ASTM standard specifies.
6. Heat treatment at 1000 °C is a promising post-treatment although it alters the microstructure, and results in a β content of ~7%, the mechanical properties; elongation, 13.7%, the YS, 927 MPa and UTS, 1063 MPa, surpass the ASTM F2924 minimum requirements. However, fatigue testing is recommended (if applicable).
7. In a future study, a model for optimizing mechanical properties and selected parameters using optimization tools and a backing mathematical model can be used in order to attain better results.

## Figures and Tables

**Table 1:** Specimen groups and their post-treatments.

No.	Group	Post Treatment
1	As-built	None
2	HIP-780	HIP, 2h @ 780 °C under 120 MPa
3	HIP-920	HIP, 2h @ 920 °C under 120 MPa
4	HT-780	Vacuum heat treatment, 2 h @ 780 °C
5	HT-1000	Vacuum heat treatment, 2 h @ 1000 °C
6	Commercial	Extruded and annealed 1/2″ rod

**Table 2:** The β phase content at room temperature of selected specimens as determined using electron backscatter diffraction (EBSD), uncertainty is ±0.5%. The average relative density of samples of the same metallurgical state is shown in the right column.

Metallurgical State	β Phase Content [%]	Relative Density [%]
As Built	<1	99.89 ± 0.03
HIP 780 °C	1.25	99.98 ± 0.02
HIP 920 °C	3.2	99.98 ± 0.04
HT 1000 °C	7	99.91 ± 0.01

**Table 3:** Energy dispersive spectroscopy (EDS) results of two spots in as-built sample (Figure 3a) and of three spots in HIP-920 sample, bright, dark and mixed area (Figure 4d).

Measured Phase	Aluminum [wt %]	Vanadium [wt %]	Iron [wt %]	Titanium [wt %]
AB—Bright Phase (1)	6.58	7.05	0.51	85.86
AB—Dark Phase (2)	6.04	5.26	0.27	88.44
HIP 920 °C—Bright Phase (1)	5.41	9.03	0.25	85.31
HIP 920 °C—Dark Phase (2)	6.60	3.25	Not detected	90.15
HIP 920 °C—Mixed Phase (3)	6.12	6.78	Not detected	87.10

**Table 4:** The Average values, standard deviations of Young’s modulus (E), 0.2% proof stress (YS), ultimate tensile strength (UTS) elongation to fracture, El., reduction of area, RA, hardness Vickers, HV10N and number of tensile tests for the various groups, typical properties on mill annealed.

Group	E * [GPa]	YS [MPa]	UTS [MPa]	El. [%]	Ra	HV_10N_	Number of Samples
AB	122.1 ± 1	1038 ± 7	1135 ± 4	7.3 ± 1.9	12 ± 5	372 ± 4	3
HT-780	120.7 ± 1	1042	1133	8.9	12	372 ± 3	1
HT-1000	118.0 ± 1	927 ± 7	1063 ± 8	13.7 ± 1.0	25 ± 5	338 ± 4	3
HIP-780	118.0 ± 1	1035 ± 1	1129 ± 1	14.4 ± 2.3	28 ± 1	374 ± 5	3
HIP-920 [4]	118.5 ± 1	976 ± 19	1090 ± 18	20.1 ± 0.7	43 ± 2	358 ± 7	5
Extruded rod-annealed	108.0 ± 1	909, 917	1034, 1037	15.4, 16.8	50 ± 1	349 ± 3	2
ASTM F136 Ti-6Al-4V Wrought		≥795 min	≥860	≥10			
ASTM F2924 Tensile		≥825	≥895	≥10			

*—Young’s modulus was calculated in the stress range 200–800 MPa, on a single sample from each batch.

**Table 5:** The number of cycles to failure, N_f_, under similar maximum stress and average stress, for various treatments.

Treatment	S_Max_ [MPa]	Number of Samples	σ_max_/YS	σ_avg_ [MPa]	Number of Cycles, N_f_
AB [4]	623	3	0.60	343	2.7 ± 1.0 × 10^4^
HIP-780	624	8	0.60	343	1.5 ± 0.4 × 10^6^
HIP-920 [4]	623	11	0.64	343	6.6 ± 3.2 × 10^6^
HIP-920 [17]	625	3	0.75	331	8.1 ± 3.2 × 10^6^

## Data Availability

Not applicable.
